# Comparative effects of unilateral and bilateral plyometric training on physical fitness in adolescent team-sport athletes: a systematic review and meta-analysis

**DOI:** 10.3389/fphys.2026.1820331

**Published:** 2026-04-27

**Authors:** Fengming Zhang, Yang Liu, Shijie Shi, Hanpeng Wang, Jiale Liu, Oleksandr Yeremenko

**Affiliations:** Department of History and Theory of Olympic Sport, National University of Physical Education and Sport of Ukraine, Kyiv, Ukraine

**Keywords:** adolescent athletes, athletic performance, meta-analysis, neuromuscular adaptation, physical fitness, plyometric training, team-sport

## Abstract

**Objective:**

This study aimed to compare the relative effects of unilateral and bilateral plyometric training on jump, sprint, and change-of-direction performance in adolescent team-sport athletes.

**Methods:**

A systematic search was conducted in PubMed, Web of Science, Scopus, and Embase databases. Effect sizes were calculated from pre- and post-training changes and pooled using a random-effects model, expressed as SMD (Hedges’ g) and its 95% CI.

**Results:**

A total of 11 studies involving 388 participants were included. Between-group comparisons showed no significant differences for countermovement jump (CMJ) (SMD = −0.06, 95% CI −0.32 to 0.20, I² = 0%, p = 0.649), standing long jump (SLJ) (SMD = −0.20, 95% CI −0.55 to 0.15, I² = 0%, p = 0.268), ≤10 m sprint (SMD = −0.22, 95% CI −0.53 to 0.09, I² = 0%, p = 0.164), ≥20 m sprint (SMD = −0.06, 95% CI −0.31 to 0.20, I² = 0%, p = 0.669), or change-of-direction (COD) (SMD = −0.01, 95% CI −0.33 to 0.31, I² = 27.9%, p = 0.946). Unilateral plyometric training showed greater improvements in single-leg tasks, including single-leg CMJ (SMD = 0.34, 95% CI 0.07 to 0.61, I² = 0%, p = 0.013) and single-leg COD (SMD = −0.61, 95% CI −1.01 to −0.21, I² = 0%, p = 0.003).

**Conclusion:**

This study found no clear between-group advantage between unilateral and bilateral plyometric training for bilateral jump performance (CMJ and SLJ), sprint performance (≤10 m and ≥20 m), or COD. However, unilateral plyometric training appeared to provide greater benefits in single-leg tasks, particularly single-leg CMJ and single-leg COD, suggesting a possible task-specific advantage.

**Systematic review registration:**

https://www.crd.york.ac.uk/prospero/, identifier PROSPERO CRD420261324213.

## 1 Introduction

Modern team sports demand more from athletes in terms of lower-limb power, sprinting, and change-of-direction, and they also require athletes to maintain movement quality and technique stability during repeated high-intensity intervals (Faude et al., 2012; Ortega-Becerra et al., 2020; Pernigoni et al., 2021). Therefore, good physical fitness supports team-sport performance by helping athletes execute skills effectively and perform at a higher level, and it may also reduce injury risk during contact situations (de la Motte et al., 2019; Ramirez-Campillo et al., 2022). Adolescents continue to undergo growth and maturation, and their neuromuscular system is highly plastic, so the choice of training methods not only affects short-term physical fitness adaptations but may also affect the development of long-term athletic ability (Lloyd and Oliver, 2012; Moran et al., 2017; Radnor et al., 2018). At the same time, physical fitness such as power, sprinting, and change-of-direction are also important criteria for selecting young team-sport talent (Barraclough et al., 2022; Burgess and Naughton, 2010; Han et al., 2023).

Notably, plyometric training (PT) has a physiological basis in the stretch-shortening cycle (SSC) and, by enhancing neuromuscular drive and the utilization of tendon elasticity, optimizes the efficiency of the eccentric-to-concentric transition, thereby improving related sport performance (Chen et al., 2023; Davies et al., 2015; Komi, 2003; Lloyd et al., 2011). Numerous studies have shown that PT can improve physical fitness such as power, speed, and change-of-direction (Asadi et al., 2016; Moran et al., 2021; Ramirez-Campillo et al., 2020). However, different support patterns (unilateral or bilateral) and directional emphasis (vertical or horizontal) will create different training stimuli, thereby affecting training adaptation and its transfer to specific sports performance (Boyle, 2016; Young, 2006). When classified by support pattern, unilateral and bilateral PT represent distinct training stimuli (Ramírez-Campillo et al., 2015). Moreover, unilateral training more closely resembles common situations in team-sport, such as single-leg support, single-leg braking, and single-leg re-acceleration, and may therefore be more sport-specific. In addition, it imposes higher demands on multi-joint neuromuscular coordination and stability and may benefit dynamic stability, unilateral strength gains, and improvements in inter-limb symmetry (Liao et al., 2022; Sun et al., 2025; Young, 2006). Bilateral training allows greater force output, which can support the development of maximal strength (Appleby et al., 2019; Juan, 2001). Under the principle of training specificity, the similarity between the movement patterns of training tasks and sport-specific skills is often considered an important factor influencing training outcomes (Loturco et al., 2015).

Although direct comparative studies of unilateral and bilateral PT have increased in recent years, the available evidence remains scattered, and findings are mixed. For example, some studies suggest that unilateral PT might be more effective at enhancing single-leg and double-leg jump performance, maximal strength, and rate of force development (Bogdanis et al., 2019; Drouzas et al., 2020). It is worth noting that many reviews and meta-analyses on plyometric training often pool different support modes, so they do not clearly show the relative differences in training effects between unilateral and bilateral programs in adolescent team-sport athletes, or the potential advantages of each (Chen et al., 2023; Lin et al., 2025; Moran et al., 2021; Ramirez-Campillo et al., 2020). In addition, existing comparative meta-analyses include mixed populations and sport contexts, including adults and adolescents as well as team and individual sports, so it is still unclear how well their conclusions apply to adolescent team-sport athletes (Zhang et al., 2025).

For these reasons, this systematic review and meta-analysis addressed the following research question: in adolescent team-sport athletes, do unilateral and bilateral PT produce different effects on countermovement jump (CMJ), standing long jump (SLJ), sprint, and change-of-direction (COD) performance? When the data permitted, subgroup analyses were planned by age, training duration, and total sessions to explore potential moderators and help explain between-study heterogeneity, thereby better informing training practice in adolescent team-sport athletes.

This article is organized into the following sections. Section 2 outlines the methods, including the search strategy, study selection, data extraction, risk-of-bias assessment, and statistical analysis. Section 3 presents the results. Section 4 discusses the key findings, practical implications, limitations, and future research needs. Section 5 summarizes the main conclusions.

## 2 Methods

This systematic review followed PRISMA 2020 and was registered in PROSPERO (CRD420261324213) (Page et al., 2021).

### 2.1 Information sources and search strategy

PubMed, Web of Science Core Collection, Scopus, and Embase were searched. Searches were run on titles and abstracts only, with no publication-year limits, and results were restricted to English. Only peer-reviewed original research articles were eligible. In addition, the reference lists of included studies and related reviews were screened to identify studies that the database search may not have captured. The full strategy is shown in **Supplementary Table 1**.

### 2.2 Eligibility criteria

Inclusion and exclusion criteria were defined using the PICOS framework, summarized in **Table 1**. Adolescence was defined as 10 to 19 years (Organization, 2023). At the same time, athlete caliber was classified using the McKay Participant Classification Framework, and only Tier ≥2 participants were included (McKay et al., 2021).

**Table 1:** Eligibility criteria.

Category	Inclusion criteria	Exclusion criteria
Population (P)	Healthy adolescent team-sport athletes aged 10–19 years, with sports including but not limited to basketball, soccer, handball, volleyball, rugby, etc.	Non-adolescents or athletes aged between 10 and 19 years Non-team sports or unclear team-sport status, Health problems or injuries that may affect training or testing, or recent surgery
Intervention (I)	Unilateral plyometric training is one of the main training interventions, consisting mainly of jump-based SSC exercises that emphasize single-leg support or single-leg take-off and landing, with a training duration of ≥4 weeks.	Training duration <4 weeks Unilateral plyometric training is not the main differentiating intervention, nor can the training content be distinguished.
Comparison (C)	Bilateral plyometric training group, mainly consisting of SSC jump exercises performed with simultaneous two-leg actions or double-leg support, included in the same study and directly compared with the unilateral group, with a training duration ≥4 weeks.	No bilateral plyometric control or comparison group Single-arm studies with unilateral-only or bilateral-only groups No direct comparison or no extractable comparable data
Outcome (O)	Reporting at least one fitness-related outcome with pre- and post-intervention data available, including jump performance (CMJ, SJ, DJ, single-leg jumps, horizontal jumps), sprint performance (≤10 m, ≥20 m, or other distances), and COD performance (505, T-test, Illinois)	Studies that did not report any fitness-related outcome measures, or studies in which pre- and post-intervention data could not be obtained
Study Design (S)	Randomized controlled trial (RCT)	Non-randomized trials, non-controlled studies, single-group pre–post designs, observational studies, and systematic reviews and meta-analyses.

### 2.3 Selection process

Author FZ used EndNote 21 to remove duplicate records. Authors YL and LJ then independently screened titles, abstracts, and full texts according to the prespecified inclusion and exclusion criteria. When disagreements arose during screening, the two authors first resolved them through discussion. If consensus could not be reached, a third author (OY) adjudicated.

### 2.4 Data extraction

Authors FZ and LJ independently extracted information from the included studies, including author and publication year, participant age and sex, sample size, sport and competitive level, training protocol, training duration and frequency, total ground contacts, and outcome measures. For each outcome, the assessment method, including the test name, measurement equipment, testing protocol, and reported reliability when available, was also extracted. Author PH checked the extracted data. If a study lacked data or had an unclear description, the study team contacted the corresponding authors to obtain the necessary information. If disagreements arose, the authors discussed the issue and reached an agreement.

### 2.5 Risk of bias assessment and certainty of evidence

Risk of bias in the included studies was assessed with the Cochrane RoB 2 tool, which covers five domains: the randomization process, deviations from intended interventions, missing outcome data, measurement of the outcome, and selection of the reported result (Sterne et al., 2019). Each domain and the overall judgment were rated as low risk, some concerns, or high risk.

The certainty of evidence was graded with the GRADE framework. Randomized controlled trials were rated as high certainty at the start. They were downgraded for risk of bias, inconsistency, indirectness, imprecision, or publication bias, with final ratings of high, moderate, low, or very low (Guyatt et al., 2011). Two authors, FZ and JS, independently completed the risk-of-bias assessment and the GRADE rating. If disagreements arose, the authors discussed the issue to reach an agreement, and a third author, OY, made the final decision when needed.

### 2.6 Statistical analysis

All statistical analyses were performed in Stata 15.0. Because baseline levels may differ across studies, effect sizes were calculated using pre-to-post change scores, defined as Mean_change_ = Mean_post_ − Mean_pre_. If an original study did not report the standard deviation of the change score, SD_change_ was estimated by assuming a pre–post correlation coefficient of r = 0.5 and using SD_change_ = √(SD_pre_² + SD_pos_t² − 2r×SD_pre_×SD_post_) (Higgins and Green, 2008). A random-effects model was used because participant characteristics, intervention protocols, and testing methods could differ across studies (Deeks et al., 2019). Effect sizes were expressed as standardized mean differences (SMD), with small-sample correction using Hedges’ g, and results were reported with 95% confidence intervals (95% CI) (Higgins and Green, 2008). Effect-size magnitudes are trivial (<0.2), small (0.2–0.6), moderate (0.6–1.2), large (1.2–2.0), very large (2.0–4.0), and extremely large (>4.0) (Hopkins et al., 2009). Between-study heterogeneity was assessed using the I2 statistic and was classified as low (<25%), moderate (25–50%), substantial (50–75%), or considerable (>75%) (Higgins and Thompson, 2002). Statistical significance was set at p < 0.05.

In the between-group analysis, the unilateral PT group served as the experimental group, and the bilateral PT group served as the control group. Within-group effect sizes were calculated from pre-to-post mean changes to describe the training effects within unilateral and bilateral PT. For time-based outcomes (sprints and COD), SMD < 0 favors unilateral PT, whereas SMD > 0 favors bilateral PT.

For single-leg outcomes, when left- and right-leg data were reported separately, they were combined into a single study-level effect size before pooling to avoid double-counting the same sample.

### 2.7 Subgroup analysis

To explore potential sources of between-study heterogeneity, subgroup analyses were planned when at least one subgroup included ≥2 studies. Moderator variables (age, training duration, total sessions) were planned to be categorized using median splits.

### 2.8 Publication bias and sensitivity analyses

Egger’s test was used to assess publication bias and small-study effects. When fewer than 10 studies were available for an outcome, the test was considered underpowered, and the results were treated as exploratory. If potential bias was indicated, the trim-and-fill method was applied. Separately, sensitivity analyses were conducted to assess the robustness of the pooled estimates.

## 3 Results

### 3.1 Study selection

**Figure 1:**
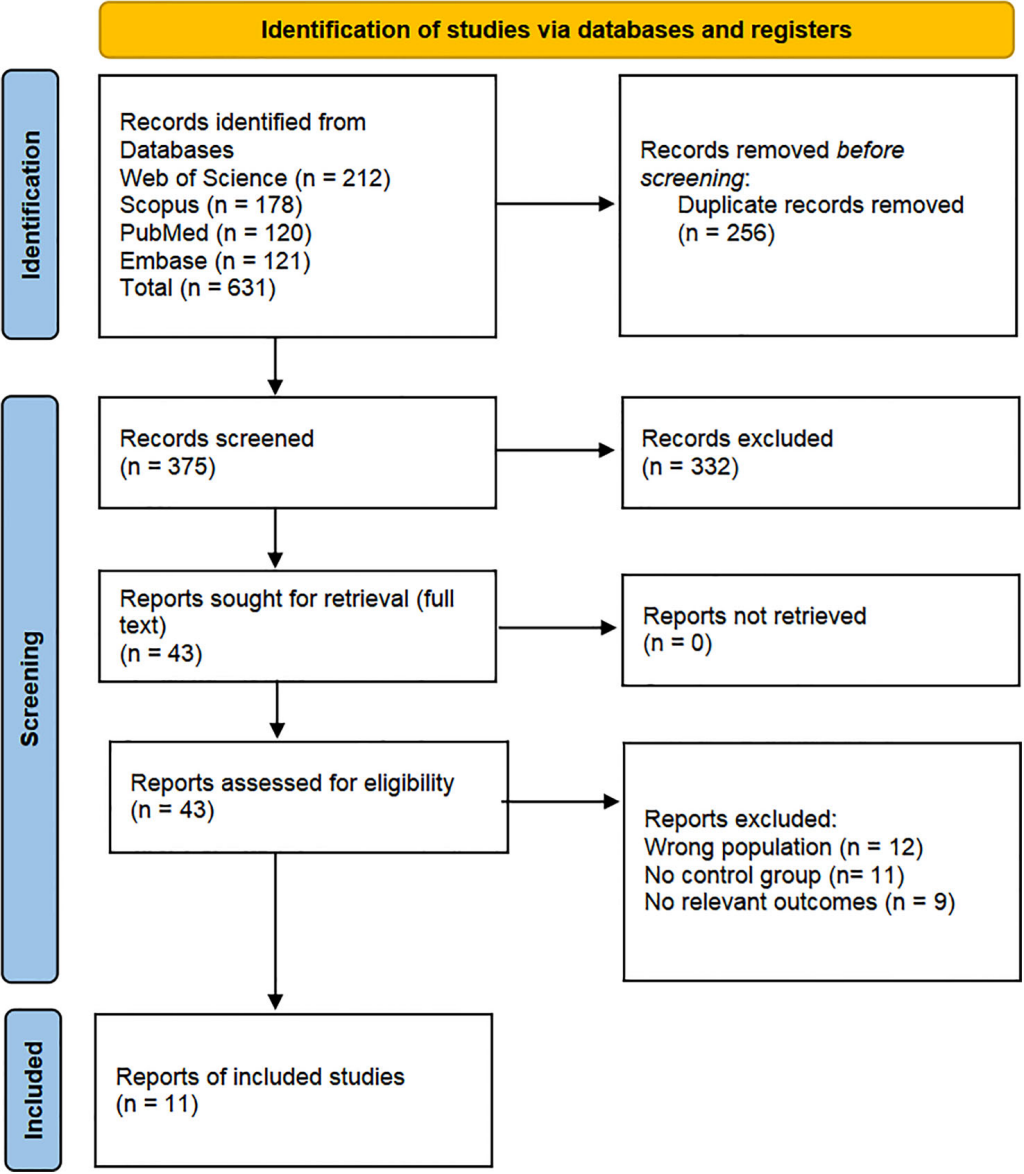
Flow diagram of the study selection process.

A total of 631 records were identified across the databases. After removing duplicates (n = 256), 375 records underwent title and abstract screening, followed by full-text review (n = 43). Ultimately, 11 studies met the inclusion criteria (Ahmad and Jain, 2020; Aztarain-Cardiel et al., 2025; Cao et al., 2024; Drouzas et al., 2020; Gonzalo-Skok et al., 2019, 2017; Hammami et al., 2025; Mujezinović et al., 2024; Stern et al., 2020; Zhang and Li, 2026; Zhao et al., 2024). **Figure 1** shows the study selection process.

### 3.2 Characteristics of participants and interventions

Study and intervention characteristics are shown in **Table 2**. In total, 11 studies were included, with 388 adolescent team-sport athletes. Most participants were male, as 10 studies included only males and one included only females. The sports represented were mainly basketball (six studies), followed by soccer (four studies) and volleyball (one study). For the competitive level, eight studies involved Tier 2 (developmental level) athletes and three involved Tier 3 (national level) athletes. For season timing, four interventions were conducted during the season, and three before the season, but the other studies did not report this item.

**Table 2:** Characteristics of participants, intervention protocols, and outcome measurements in the included studies.

Studies	Participants characteristics	Sport	Characteristics of intervention						Measurements
			Season	Duration (weeks)	Frequency (sessions)	Total sessions (n)	Total ground contacts (n)	Training protocol	
Gonzalo-Skok et al., 2017	TB:2-5; S: M EG1: N = 11, A = 16.8 ± 1.7; EG2: N = 11, A = 16.7 ± 1.7; PL: National (Tier 3)	Basketball	In-season	6	2	12	480	Unilateral drop jumps and Unilateral countermovement jump (left and right legs) EG2: Bilateral drop jumps and Bilateral countermovement jump	CMJ, 5m, 25m, 25m Sprint, COD (V-cut)
Gonzalo-Skok et al., 2019	TB: NR; S: M EG1: N = 9, A = 13.2 ± 0.5; EG2: N = 9, A = 13.3 ± 0.6; PL: Developmental (Tier 2)	Basketball	In-season	6	2	12	960	EG1: Unilateral-horizontal PT (left and right legs, Drop Jump 10 cm, Standing long jump (SLJ), SLJ without countermovement, unilateral jumps, triple jumps) EG2: Bilateral-vertical PT (Drop jump 20 cm, Squat jump with arm swing, Countermovement jump with arm swing, Tuck jump, Hurdle jumps)	CMJ, 5m, 15m, 25m Sprint, COD (V-cut, 180)
Ahmad and Jain, 2020	TB: NR; S: FM EG1: N = 33, A = 16.16 ± 1.65; EG2: N = 33, A = 16.18 ± 1.80; PL: Developmental (Tier 2)	Volleyball	NR	8	2	16	NR	EG1: Unilateral PT program EG2: Bilateral PT program	CMJ, SJ, SLJ, COD (T-test)
Drouzas et al., 2020	S: M EG1: N = 23, A = 9.9 ± 1.8, TB = 4.3 ± 2.0; EG2: N = 23, A = 10.0 ± 0.5, TB = 3.5 ± 1.5; PL: Developmental (Tier 2)	Soccer	In-season	8	2	16	1440	EG1: EG1: EG1: Unilateral jumps in squares, unilateral hurdle jumps, unilateral lateral jumps, unilateral ladder jumps, unilateral diagonal jumps, unilateral step jumps (left and right legs) EG2: bilateral jumps in squares, bilateral hurdle jumps, bilateral lateral jumps, bilateral ladder jumps, bilateral diagonal jumps, bilateral step jumps	CMJ, SJ, SLJ, 5m, 10m, 20m sprint, COD (T-test)
Stern et al., 2020	TB: ≥2; S: M; A = 17.6 ± 1.2 EG1: N = 11, EG2: N = 11; PL: National (Tier 3)	Soccer	Pre-season	6	2	12	576	EG1: Single-leg drop jump, Single-leg countermovement jump, Single-leg broad jump (left and right legs) EG2: Drop jump, Countermovement jump, Broad jump	CMJ, SLJ, 10m, 30m sprint, SCOD
Cao et al., 2024	TB: ≥2; S: M EG1: N = 16, A = 15.9 ± 0.9; EG2: N = 16, A = 16.3 ± 0.8; PL: Developmental (Tier 2)	Basketball	NR	8	2	16	1000	EG1: Unilateral horizontal jump, unilateral 3-bounce jumps, unilateral reactive pogo jumps, unilateral countermovement jumps, unilateral drop jumps (10 cm) (left and right legs)	SCOD(5-0–5 test)
Mujezinović et al., 2024	TB: ≥2; S: M; A: 14 EG1: N = 15, EG2: N = 15; PL: National (Tier 3)	Soccer	NR	8	3	24	NR	EG1: Unilateral PT (left and right legs) single-leg jumps EG2: Bilateral PT two-leg jumps	5m, 20m sprint, COD(5-0–5 test)
Zhao et al., 2024	TB: ≥3; S: M EG1: N = 25, A = 16.2 ± 0.6; EG2: N = 25, A = 16.4 ± 0.6; PL: Developmental (Tier 2)	Basketball	NR	8	2	16	1000	EG1: Unilateral horizontal jump, unilateral 3-bounce jumps, unilateral reactive pogo jumps, unilateral countermovement jumps, unilateral drop jumps (10 cm) (left and right legs) EG2: Bilateral horizontal jumps, bilateral three horizontal jumps, bilateral reactive pogo jumps, bilateral countermovement jumps, bilateral drop jumps (10 cm)	CMJ, SJ
Aztarain-Cardiel et al., 2025	TB: 6.4 ± 1.0; S: M EG1: N = 15, A = 14.1 ± 1.3; EG2: N = 15, A = 14.2 ± 1.1; PL: Developmental (Tier 2)	Basketball	In-season	6	2	12	592	EG1: Vertical acyclic and Horizontal acyclic (left and right legs) EG2: Vertical acyclic and Horizontal acyclic	CMJ, SLJ 20m sprint, COD (V-cut)
Hammami et al., 2025	TB: 2-2.5; S: M; A=12.03 ± 2.34 EG1: N = 12; EG2: N = 12; EG3: N = 11; EG4: N = 11; PL: Developmental (Tier 2)	Soccer	Pre-season	8	2	16	1728	EG1 and EG3: Unilateral 20-cm drop jump, unilateral horizontal jumps, unilateral lateral hops (left and right legs) EG2 and EG4: 20-cm drop jump, horizontal jumps, lateral hops	20m sprint, COD (15m)
Zhang and Li, 2026	TB: ≥3; S: M EG1: N = 13, A = 15.36 ± 1.43; EG2: N = 13, A = 16.27 ± 1.10; PL: Developmental (Tier 2)	Basketball	Pre-season	8	2	16	NR	EG1: Single-leg hops, bounds, low box step-offs, single-leg vertical hops, single-leg lateral hops, alternate bounds, single-leg drop jumps (left and right legs) EG2: Squat jumps, double-leg bounds, drop jumps, tuck jumps, depth jumps, complex plyometric sequences	CMJ, SLJ, 10m, 20m sprint, COD(5-0–5 test)

A, age; F, female; M, male; TB, training background; NR, not reported; EG, experimental group; CMJ, countermovement jump; SJ, squat jump; SLJ, standing long jump; COD, change of direction; SCMJ, Single-leg countermovement jump; SCOD, Single-leg change of direction; PL: athlete level as originally reported in the included studies; McKay tier, athlete caliber classified using the McKay Participant Classification Framework (PCF; Tier 0–5).

For the interventions, the duration was 6 to 8 weeks. Most studies used 2 sessions per week, and only 1 used 3, so the total sessions ranged from 12 to 24. Total ground contacts ranged from 480 to 1,728.

Detailed outcome assessment procedures, including test protocols, measurement equipment, and reported reliability where available, are provided in **Supplementary Table 2**.

### 3.3 Risk of bias assessment and certainty of evidence

**Figure 2:**
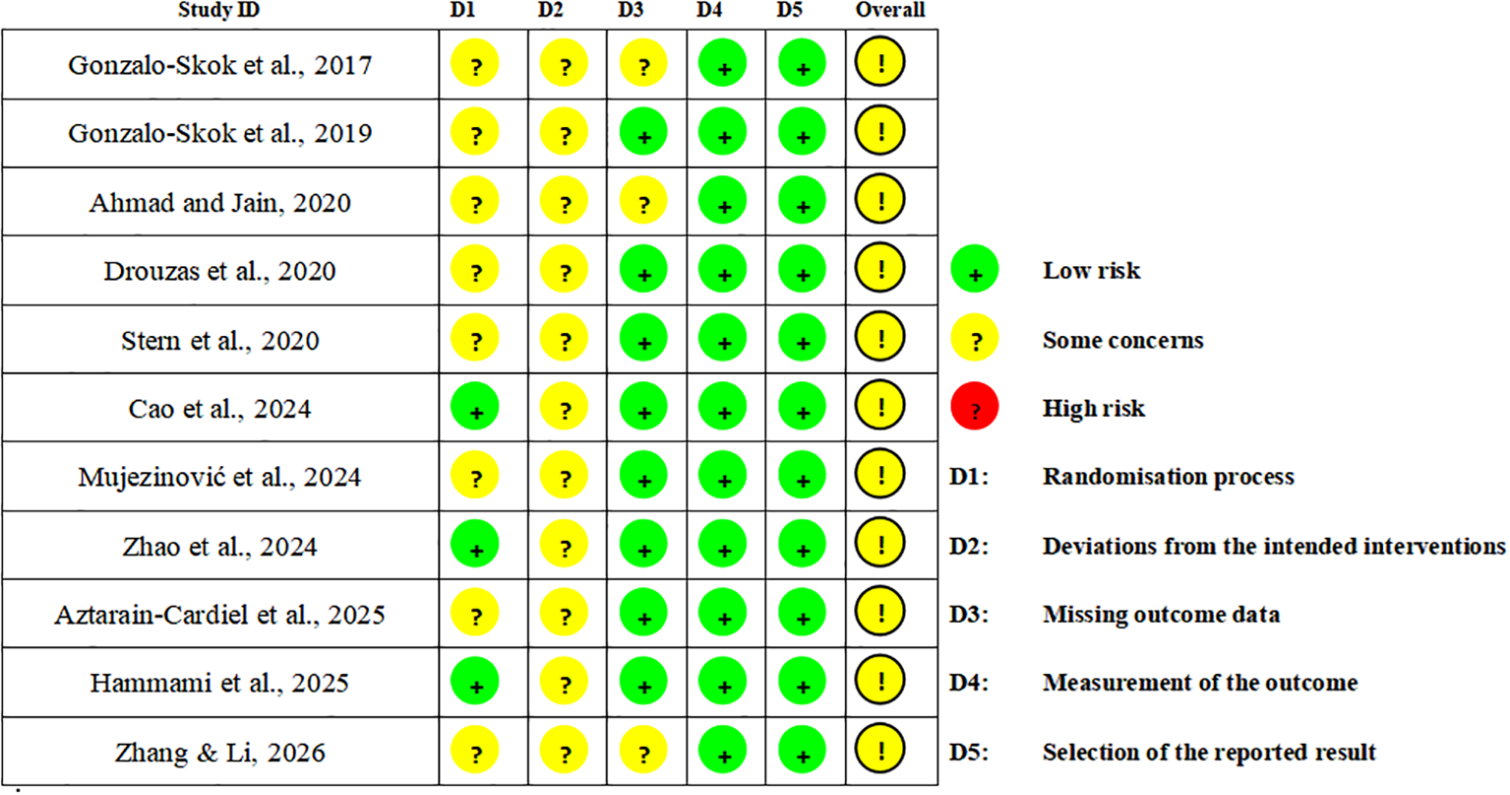
RoB 2 assessments.

**Figure 3:**
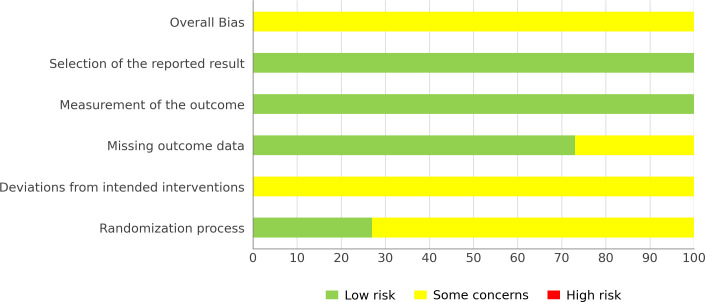
Risk of overall bias.

**Figures 2**, **3** present the RoB 2 assessment results. Overall, the risk of bias across all 11 studies was rated as “some concerns”. The main sources of risk of bias were concentrated in the “randomization process” and “deviations from intended interventions”. For the “randomization process” domain, only 3 studies reported information on allocation concealment, whereas the remaining studies either did not provide this information or reported it inadequately. For the “deviations from intended interventions” domain, all studies were rated as “some concerns”, mainly because blinding of participants and personnel is difficult to implement in exercise training interventions, which may increase the likelihood of deviations and the uncertainty in assessing their impact. For the “missing outcome data” domain, three studies were rated as “some concerns”, mainly because some participants did not complete testing or because the sample size included in the final analysis was reduced.

All included studies were randomized controlled trials, so the starting level of evidence was high. The GRADE assessment indicated that the certainty of evidence ranged from low to moderate. Except for the ≥20 m sprint, which was rated as moderate, CMJ, SLJ, single-leg CMJ, ≤10 m sprint, COD, and single-leg COD were all rated as low. Downgrading was mainly due to the risk of bias and imprecision. Because most outcomes included fewer than 10 studies, tests for publication bias were underpowered. Therefore, the certainty of the evidence was not downgraded due to publication bias. Details are shown in **Table 3**.

**Table 3:** GRADE analysis.

Certainty assessment							
Number of studies (Participants)	Study design	Risk of bias	Inconsistency	Indirectness	Imprecision	Other considerations	Quality
CMJ							
7(230)	RCT	Serious^a^	Not serious	Not serious	Serious^c^	NO^d^	⨁⨁◯◯ low
SLJ							
4(124)	RCT	Serious^a^	Not serious	Not serious	Serious^c^	NO^d^	⨁⨁◯◯ low
Single-leg CMJ							
6(214)	RCT	Serious^a^	Not serious	Not serious	Serious^c^	NO^d^	⨁⨁◯◯ low
≤10-m sprint							
6(164)	RCT	Serious^a^	Not serious	Not serious	Serious^c^	NO^d^	⨁⨁◯◯ low
≥20-m sprint							
8(240)	RCT	Serious^a^	Not serious	Not serious	Not serious	NO^d^	⨁⨁⨁◯ Moderate
Change-of-direction							
7(218)	RCT	Serious^a^	Not serious	Not serious	Serious^c^	NO^d^	⨁⨁◯◯ low
Single-leg change-of-direction							
4(106)	RCT	Serious^a^	Not serious	Not serious	Serious^c^	NO^d^	⨁⨁◯◯ low

^a^ Some included studies had methodological limitations. ^b^There was significant heterogeneity in the effect sizes observed across studies. ^c^The confidence intervals for the effect estimates for multiple outcome indicators were too wide, and the total sample size for some outcomes was lower than that required to reliably detect the true effect. ^d^Small study effects assessed, but K<10, no downgrade for publication bias.

### 3.4 Meta-analysis results

**Figure 4:**
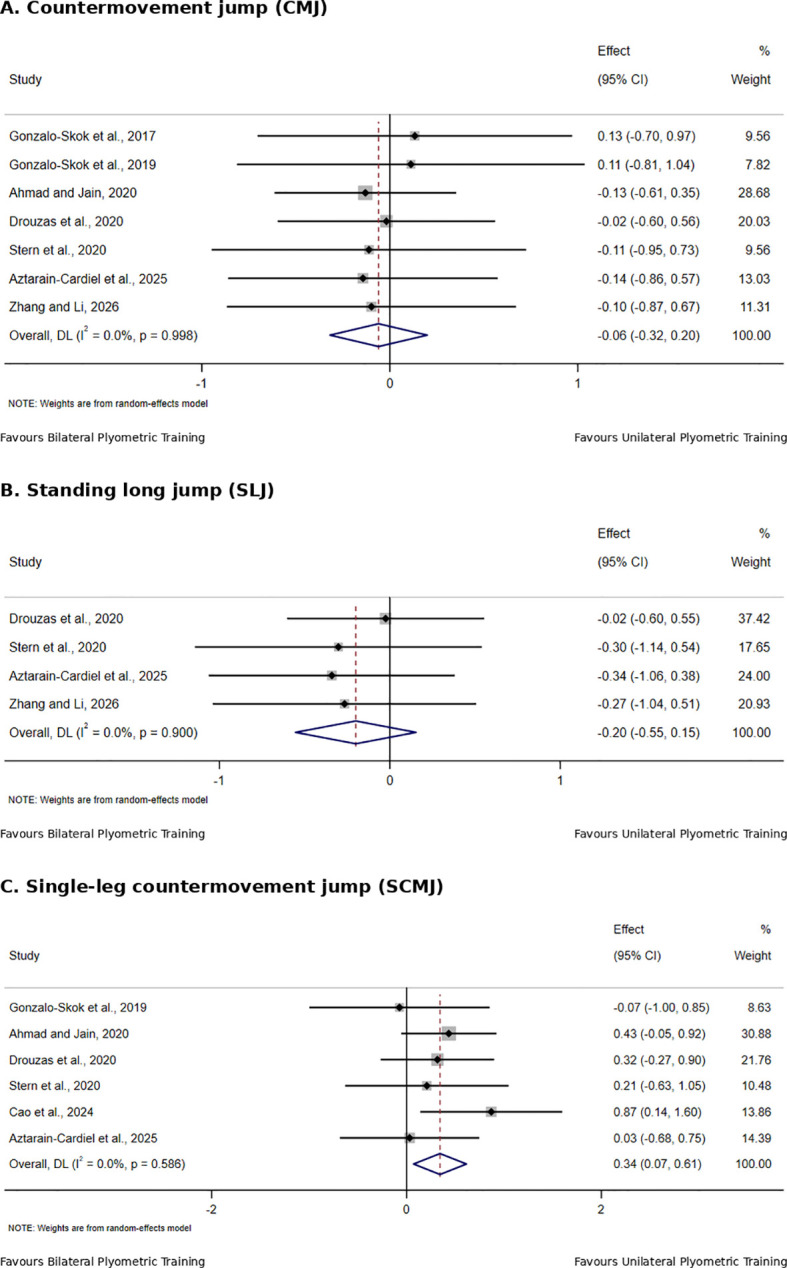
Effects of unilateral versus bilateral plyometric training on jump in adolescent team-sport athletes.

**Figure 5:**
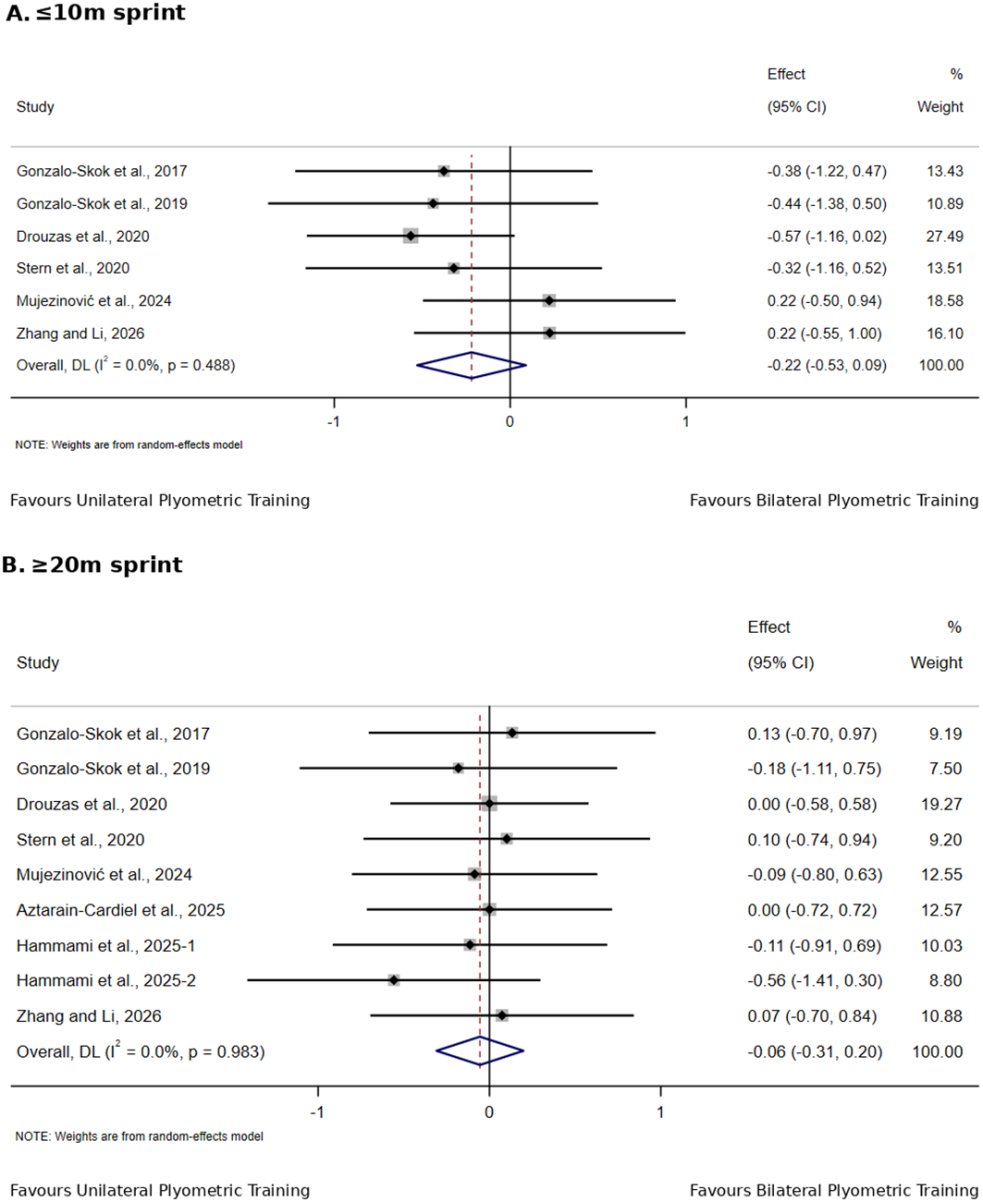
Effects of unilateral versus bilateral plyometric training on sprint in adolescent team-sport athletes.

**Figure 6:**
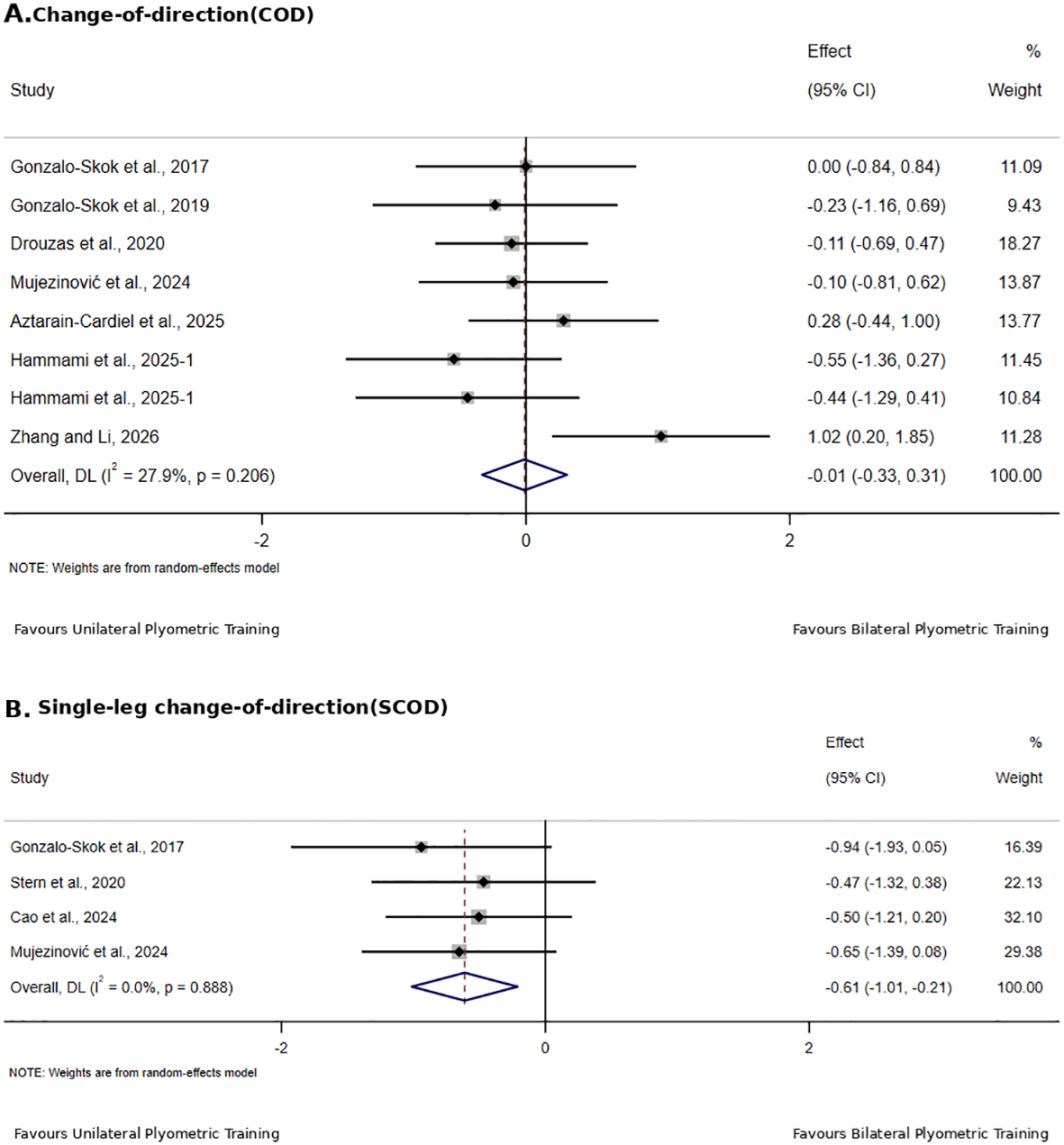
Effects of unilateral versus bilateral plyometric training on change-of-direction in adolescent team-sport athletes.

**Table 4** summarizes the direct comparisons between unilateral and bilateral PT for physical fitness outcomes in adolescent team-sport athletes, with the corresponding forest plots shown in **Figures 4**–**6**.

**Table 4:** Pooled effects of unilateral versus bilateral plyometric training on physical fitness outcomes.

Outcome	K,n	SMD (95%CI)	p (Overall Effect)	I^2^ (%)	RW (%)	Egger’s test (p)
Countermovement jump	7,230	-0.06 (-0.32 to 0.20)	0.649	0	7.82-28.68	0.216
Standing long jump	4,124	-0.20 (-0.55 to 0.15)	0.268	0	17.65-37.42	0.123
Single-leg countermovement jump	6,214	0.34 (0.07 to 0.61)	0.013	0	8.63-30.88	0.487
≤10-m sprint	6,164	-0.22 (-0.53 to 0.09)	0.164	0	10.89-27.49	0.755
≥20-m sprint	8,240	-0.06 (-0.31 to 0.20)	0.669	0	7.50-19.27	0.440
Change-of-direction	7,218	-0.01 (-0.33 to 0.31)	0.946	27.9	9.43-18.27	0.987
Single-leg change-of-direction	4,106	-0.61 (-1.01 to -0.21)	0.003	0	16.39-32.10	0.332

K: Data denote the number of studies that provided data for the analysis. n: The total number of adolescent team sport players included in the analysis, respectively.

For bilateral jump performance, CMJ included seven studies with 230 participants, showing a non-significant pooled effect with no heterogeneity (SMD = −0.06, 95% CI −0.32 to 0.20, I² = 0%, p = 0.649). SLJ included four studies with 124 participants, with no significant between-group difference (SMD = −0.20, 95% CI −0.55 to 0.15, I² = 0%, p = 0.268).

For single-leg jump performance, single-leg CMJ included six studies with 214 participants, and the results indicated a greater improvement with unilateral PT, with no heterogeneity across studies (SMD = 0.34, 95% CI 0.07 to 0.61, I² = 0%, p = 0.013).

For sprint performance, the ≤10 m sprint included six studies with 164 participants; the effect favored unilateral PT, but the between-group difference did not reach statistical significance (SMD = −0.22, 95% CI −0.53 to 0.09, I² = 0%, p = 0.164). ≥20 m sprint included eight studies with 240 participants, with no significant between-group difference (SMD = −0.06, 95% CI −0.31 to 0.20, I² = 0%, p = 0.669).

For COD, seven studies with 218 participants were included; the pooled result showed no significant between-group difference, with low heterogeneity (SMD = −0.01, 95% CI −0.33 to 0.31, I² = 27.9%, p = 0.946).

Single-leg COD included 4 studies with 106 participants; results indicated greater improvement with unilateral PT, with no heterogeneity across studies (SMD = −0.61, 95% CI −1.01 to −0.21, I² = 0%, p = 0.003).

The within-group pooled effects suggested that both unilateral and bilateral PT improved most physical fitness outcomes after the intervention, with detailed results reported in **Supplementary Tables 3**, **4**.

Exploratory subgroup analyses by age and intervention duration were conducted where possible. However, the small number of studies within subgroups limited statistical power, and no consistent subgroup pattern was observed across outcomes. Total sessions was not presented separately because it largely overlapped with intervention duration. Therefore, these exploratory analyses should not be interpreted as robust evidence regarding potential moderators. Detailed subgroup results are provided in **Supplementary Table 5**.

### 3.5 Publication bias and sensitivity analyses

Given the limited number of studies included for most outcomes, the Egger’s test was used only to explore publication bias and small-study effects, and the related results should be interpreted with caution. The results are shown in **Table 4**. The leave-one-out sensitivity analysis showed that, after removing any single study, the direction of the pooled effect size and the main conclusions did not change materially across outcomes, which suggested that the overall results were robust. Details are provided in **Supplementary Figures 1**-**7**.

## 4 Discussion

This systematic review compared the relative effects of unilateral and bilateral plyometric training on physical fitness outcomes in adolescent team-sport athletes. Overall, the direct comparisons did not show consistent evidence of a between-group difference in CMJ, SLJ, ≤10 m sprint, ≥20 m sprint, or COD. The main differences were observed in single-leg tasks, where improvements in single-leg CMJ and single-leg COD tended to favor unilateral plyometric training. However, the certainty of evidence was low for most outcomes, most comparisons were based on a limited number of studies, and most included studies had methodological limitations, particularly in the randomization process, deviations from intended interventions, and inadequate reporting of allocation concealment. This may have reduced baseline comparability between groups and obscured true between-group differences. Therefore, these findings should not be interpreted as evidence that unilateral and bilateral PT are equally effective, and the apparent advantage of unilateral PT for single-leg outcomes should not be considered conclusive.

### 4.1 Jump performance

For CMJ and SLJ, the pooled results did not show consistent evidence of a between-group difference. These findings suggest no clear advantage of either unilateral or bilateral PT for bilateral jump tasks. The pooled within-group results also indicated that both approaches improved bilateral jump performance. In general, PT may enhance jump performance through several neuromuscular adaptations, including greater neural drive, increased tendon stiffness, and more effective use of elastic energy, changes in muscle architecture, improved intermuscular and intramuscular coordination, and heightened stretch-reflex excitability (Markovic, 2007; Markovic and Mikulic, 2010; Seiberl et al., 2021).

The absence of a significant between-group difference in bilateral jump outcomes may have several explanations. As the adolescent neuromuscular system continues to mature, tendon and joint stiffness, motor unit recruitment efficiency, and stretch reflex regulation can improve with growth, thereby raising SSC function in both training modes (Radnor et al., 2018). Adolescents also show high plasticity to training stimuli, and PT may further improve the use of elastic energy and rapid activation, in addition to developmental gains (Lambertz et al., 2003; Lloyd et al., 2011). As a result, the combined effects of development and training adaptation may bring the between-group responses closer. In addition, both unilateral and bilateral PT provide high-intensity SSC and rapid force-production stimuli, so similar effects are more likely for bilateral jump tasks such as CMJ and SLJ.

It is also worth noting that the clearer differences were mainly observed in single-leg jump outcomes. Although single-leg CMJ favored unilateral PT, the pooled effect size was small (SMD = 0.34). This may reflect the greater movement specificity of unilateral PT, as it more closely matches the single-leg support, braking, and coordination demands of the single-leg CMJ (Barrio et al., 2024; Chijimatsu et al., 2020; Drouzas et al., 2020; Spudić et al., 2025).Therefore, this finding may be better interpreted as a task-specific advantage rather than a clear overall superiority of unilateral PT. From a practical perspective, this suggests that unilateral PT may be particularly useful when the training goal is to improve single-leg jump ability or other actions requiring force production under unilateral support, but it does not necessarily justify replacing bilateral PT in general programming.

### 4.2 Sprint performance

Across the comparative analyses, no consistent evidence of a between-group difference was observed for ≤10 m or ≥20 m sprint performance. The pooled within-group analysis also indicated improvements in both sprint distances after each approach. These gains may have been supported by improved neuromuscular drive, better intramuscular and intermuscular coordination, and greater overall lower limb stiffness after PT (Mackala and Fostiak, 2015; Rimmer and Sleivert, 2000; Tomalka et al., 2020). These changes can shorten ground contact time, increase stride frequency, and thus improve sprint speed (Mackala and Fostiak, 2015). In addition, sprinting is a typical SSC-dominant task, and the plyometric-induced improvement in SSC utilization aligns closely with the kinematic demands of sprinting (Morin et al., 2012).

It is worth noting that unilateral PT showed a small favorable trend for ≤10 m sprint, but the difference was not significant (p > 0.05). Compared with bilateral PT, unilateral PT more closely matches the single-leg support and single-leg force demands of the sprint start, places greater emphasis on stability control under single-leg conditions and on force transmission and coordination, and may improve the coupling efficiency between eccentric braking and concentric propulsion. As a result, a small advantage may be more likely during the start and the first few steps of early acceleration (Drouzas et al., 2020; Katsuge et al., 2025; Liao et al., 2022; Zhang et al., 2023). However, this advantage is only a trend and should be interpreted with caution, and larger direct-comparison studies are still needed.

The core of sprint performance lies in rapid hip, knee, and ankle during the starting phase, enabling athletes to achieve high levels of force output and accelerate rapidly in a very short time. At the same time, the sprint process also relies on rapid transitions between the swing and support phases, as well as on control of stride length and stride frequency, and of trunk and pelvic posture (Bezodis et al., 2019; Hunter et al., 2004; Murphy et al., 2003). Therefore, if the intervention relied mainly on unilateral or bilateral PT to improve neural drive and stretch–shortening cycle (SSC) efficiency but did not adequately integrate training of key sprint technical elements, this strategy may not have been sufficient to produce meaningful between-group differences over the short term. Sprint performance also shows phase-specific force-direction demands, as acceleration relies more on horizontal force, whereas running near maximal speed relies more on vertical force. Accordingly, plyometric programs that emphasize different force directions may have influenced sprint outcomes at short and longer distances differently. If the training plan did not provide both horizontal and vertical force stimuli, this mismatch may also have diluted any relative advantage between the two methods (Junge et al., 2023; Moran et al., 2021; Morin et al., 2011).

### 4.3 Change-of-direction

The available evidence did not show a consistent between-group difference in COD between unilateral and bilateral PT. The within-group pooled results indicated that both PT methods improved COD. Good COD ability is essential in team sports such as basketball, soccer, and handball, as it not only supports athletes’ attacking and defending and the creation of scoring opportunities, but may also help reduce sports injury risk (Dos’ Santos et al., 2017, 2019). Multiple factors influence COD and depend largely on deceleration and braking capacity, rapid eccentric-to-concentric transition ability, lower-limb stiffness regulation, and multi-joint coordinative control (Dos’ Santos et al., 2017; Sasaki et al., 2011). During COD, the lower-limb muscles must rapidly transition from eccentric braking to concentric propulsion during a short period of ground contact (Nygaard Falch et al., 2019). In the concentric phase, re-acceleration calls for a strong propulsive push in a moment, but in the eccentric phase, the athlete relies more on eccentric strength to take up the impulse and slow down (Chaabene et al., 2018; Sheppard and Young, 2006). PT may enhance SSC braking-to-propulsion transition efficiency by increasing eccentric strength and deceleration capacity during the braking phase, enhancing neuromuscular drive, and improving intra- and inter-muscular coordination. It may also improve balance and joint stability, thereby optimizing posture and center-of-mass control during cutting (Asadi et al., 2016; Markovic and Mikulic, 2010; Yeadon et al., 2010). These factors together promote improvements in COD performance. Given that adolescents show greater plasticity in neuromuscular adaptation and that both programs may induce convergent neuromuscular adaptations, COD may be unlikely to show stable between-group differences over the short term.

Notably, unilateral PT showed a statistically significant advantage in single-leg COD performance, which may be closely related to the principle of training specificity. The single-leg COD test mainly reflects how the stance limb performs during a single cut: it breaks to decelerate, executes the direction change during ground contact, and then re-accelerates after the cut (Clemente et al., 2022; Nimphius et al., 2016; Taylor et al., 2019). This more closely matches team-sport contexts. Unilateral PT places greater emphasis on trunk and pelvic control under single-leg support, hip–knee–ankle coordination, and rapid coupling between single-leg braking impulse and propulsive impulse, which may transfer more readily to single-leg COD tasks and thus manifest a clearer between-group advantage (Cao et al., 2024; Li et al., 2025; Zhang et al., 2023). The magnitude of this effect was moderate (SMD = −0.61), suggesting greater practical relevance than the small effect observed for single-leg CMJ. For coaches, this may indicate that unilateral PT deserves greater emphasis when the training priority is to enhance cutting actions performed from unilateral support, particularly those involving braking, re-acceleration, and directional changes that closely resemble team-sport demands. However, given the limited number of studies and low certainty of evidence, this implication should still be applied with caution and confirmed in future studies.

### 4.4 Study limitations

Several limitations should be considered when interpreting the findings of this study. First, the number of included studies was limited, and the sample sizes for most physical fitness outcomes were small, which may have constrained the precision of the pooled effect estimates. The certainty of evidence was low for most outcomes, which reduces confidence in the findings and weakens the overall strength of the conclusions. In addition, several studies provided insufficient information on randomization procedures and allocation concealment, which further reduces confidence in the pooled estimates. Second, there was a marked sex imbalance, with participants predominantly male adolescent team-sport athletes, which limits the generalizability of the conclusions to female adolescent team-sport athletes. At the same time, the included sports were mainly soccer and basketball, which also limits the applicability of the conclusions to other team sports such as handball and volleyball. Third, intervention heterogeneity should also be considered. Variability across studies in exercise selection, particularly the relative emphasis on vertical versus horizontal plyometric tasks, total ground contacts, and session structure, may have attenuated true between-group differences. These intervention characteristics were not formally modeled because of the limited number of available studies, which also restricts the interpretation of subgroup findings and potential moderators. In the statistical analysis, when some studies did not report the SD of change scores, a fixed correlation coefficient was used for estimation, and for single-leg outcomes, left- and right-leg data were combined to avoid double-counting. These necessary assumptions may have influenced the effect size estimates. Finally, although exploratory subgroup analyses by age and intervention duration were conducted where possible, the small number of studies within subgroups limited statistical power. Therefore, moderator-related factors such as age, maturity, and training dose could not be examined robustly, and these moderator-related findings should be interpreted cautiously.

### 4.5 Future research directions

Future studies should include more balanced sex representation to improve the applicability of the findings to both male and female adolescent team-sport athletes. In addition, broader sport sampling is needed, particularly in team sports that remain underrepresented in the current evidence base, such as handball, rugby, and volleyball. Longer interventions are also needed, together with dose-response trials that systematically vary total ground contacts, session frequency, and the relative emphasis on vertical versus horizontal plyometric tasks. In addition, clearer reporting of training content, load progression, and outcome assessment procedures would further strengthen comparability across studies and improve the interpretability of pooled findings.

### 4.6 Practical applications

In practice, both unilateral and bilateral PT appear to be feasible and effective options for improving jumping, sprinting, and COD performance in adolescent team-sport athletes. When the training goal is to enhance single-leg power, single-leg COD, start acceleration, or other actions that rely heavily on unilateral support, coaches may place greater emphasis on unilateral plyometric exercises, while still retaining bilateral exercises as part of the overall program. It may be considered to adopt a training duration of 6 to 8 weeks, with a training frequency of 2 times per week as the approach, and to make individual adjustments based on the athlete’s training foundation, fatigue monitoring, and recovery status.

## 5 Conclusion

This study did not show consistent evidence of a between-group difference between unilateral and bilateral plyometric training for bilateral jump performance (CMJ and SLJ), sprint performance (≤10 m and ≥20 m), or COD in adolescent team-sport athletes. However, unilateral plyometric training tended to favor single-leg tasks, particularly single-leg CMJ and single-leg COD, suggesting a possible task-specific advantage. Overall, both training modes may be useful options for improving physical fitness performance, and the proportion of unilateral PT may be increased within the overall training program when the primary training goal is to improve single-leg performance or actions that rely heavily on unilateral support.

## Data Availability

The datasets presented in this study can be found in online repositories. The names of the repository/repositories and accession number(s) can be found in the article/**Supplementary Material**.
