# Supplementary material for: Comparative effects of unilateral and bilateral plyometric training on physical fitness in adolescent team-sport athletes: a systematic review and meta-analysis
Source: Front Physiol. 2026 Apr 27;17:1820331. doi: 10.3389/fphys.2026.1820331 (PMC13158094; doi:10.3389/fphys.2026.1820331)
Supplement: Supplementary file 1 [file Table1.docx]

Table S1. Full electronic search strategies for all databases

| **Database** | **Complete Search Strategy** | **Hits** |
| --- | --- | --- |
| Web of Science | TS=(plyometric* OR "jump training" OR "stretch shortening cycle" OR SSC OR "reactive strength" OR "drop jump" OR "depth jump" OR "countermovement jump" OR CMJ OR hopping OR bounding OR skipping)  AND TS=(unilateral* OR "single leg" OR "single legged" OR "single-legged" OR "single limb" OR "unilateral jump*" OR "single leg jump*")  AND TS=(bilateral* OR "double leg" OR "double limb" OR "two limb" OR "bilateral jump*")  AND TS=("team sport*" OR soccer OR football OR basketball OR handball OR volleyball OR rugby) | 212 |
| Scopus | TITLE-ABS-KEY(plyometric* OR "jump training" OR "stretch shortening cycle" OR SSC OR "reactive strength" OR "drop jump" OR "depth jump" OR "countermovement jump" OR CMJ OR hopping OR bounding OR skipping)  AND TITLE-ABS-KEY(unilateral* OR "single leg" OR "single legged" OR "single-legged" OR "single limb" OR "unilateral jump*" OR "single leg jump*")  AND TITLE-ABS-KEY(bilateral* OR "double leg" OR "double limb" OR "two limb" OR "bilateral jump*")  AND TITLE-ABS-KEY("team sport*" OR soccer OR football OR basketball OR handball OR volleyball OR rugby) | 178 |
| PubMed | (plyometric*[tiab] OR "jump training"[tiab] OR "stretch shortening cycle"[tiab] OR SSC[tiab] OR "reactive strength"[tiab] OR "drop jump"[tiab] OR "depth jump"[tiab] OR "countermovement jump"[tiab] OR CMJ[tiab] OR hopping[tiab] OR bounding[tiab] OR skipping[tiab])  AND (unilateral*[tiab] OR "single leg"[tiab] OR "single legged"[tiab] OR "single-legged"[tiab] OR "single limb"[tiab] OR "unilateral jump*"[tiab] OR "single leg jump*"[tiab])  AND (bilateral*[tiab] OR "double leg"[tiab] OR "double limb"[tiab] OR "two limb"[tiab] OR "bilateral jump*"[tiab])  AND ("team sport*"[tiab] OR soccer[tiab] OR football[tiab] OR basketball[tiab] OR handball[tiab] OR volleyball[tiab] OR rugby[tiab]) | 120 |
| Embase | (plyometric*:ti,ab,kw OR "jump training":ti,ab,kw OR "stretch shortening cycle":ti,ab,kw OR SSC:ti,ab,kw OR "reactive strength":ti,ab,kw OR "drop jump":ti,ab,kw OR "depth jump":ti,ab,kw OR "countermovement jump":ti,ab,kw OR CMJ:ti,ab,kw OR hopping:ti,ab,kw OR bounding:ti,ab,kw OR skipping:ti,ab,kw)  AND (unilateral*:ti,ab,kw OR "single leg":ti,ab,kw OR "single legged":ti,ab,kw OR "single-legged":ti,ab,kw OR "single limb":ti,ab,kw OR "unilateral jump*":ti,ab,kw OR "single leg jump*":ti,ab,kw)  AND (bilateral*:ti,ab,kw OR "double leg":ti,ab,kw OR "double limb":ti,ab,kw OR "two limb":ti,ab,kw OR "bilateral jump*":ti,ab,kw)  AND ("team sport*":ti,ab,kw OR soccer:ti,ab,kw OR football:ti,ab,kw OR basketball:ti,ab,kw OR handball:ti,ab,kw OR volleyball:ti,ab,kw OR rugby:ti,ab,kw) | 121 |

Table S2. Detailed outcome assessment procedures across included studies

| **Study** | **Outcome** | **Test name** | **Equipment** | **Protocol / scoring rule** | **Reliability reported** |
| --- | --- | --- | --- | --- | --- |
| Gonzalo-Skok et al. 2017 | CMJ, 5m, 25m, 25m Sprint, COD (V-cut) | Countermovement jump 25-m linear sprint V-Cut | Optojump (Microgate) Microgate photoelectric cells / timing gates | Sprint: standing start, front foot 0.5 m before first gate; 2 trials, ≥3-min rest, best time. CMJ: hands on hips, self-selected depth, 3 trials, 45-s rest, best jump. V-Cut: 25 m with 4 x 45° turns every 5 m; 2 trials, 3-min rest, best time. | Within-study ICC and CV reported for sprint, CMJ, V-Cut. |
| Gonzalo-Skok et al. 2019 | CMJ, 5m, 15m, 25m Sprint, COD (V-cut) | Countermovement jump 5m, 15m, 25m sprint V-Cut | Optojump (Microgate) Witty/Microgate timing gates Tape measure for HJ | Sprint: start 0.5 m before first gate; 2 trials,≥3 min rest, best time. CMJ: hands on hips, 3 trials, 45-s rest, best jump. Unilateral CMJ: single-leg trials with controlled posture; best of 3. V-Cut: 25 m with 4 x 45° turns, 2 trials, 3-min rest, fastest. | Within-study ICC and CV reported for sprint, CMJ, V-Cut. |
| Ahmad  2020 | CMJ, SJ, SLJ, COD (T-test) | Countermovement vertical jump Squat jump Standing long jump T-agility test | NR | NR | NR |
| Drouzas et al.  2020 | CMJ, SJ, SLJ, 5m, 10m, 20m sprint, COD (T-test) | Countermovement jump Squat jump Standing broad jump  Linear sprint Modified T-test | Chronojump platform for vertical jumps Fitlight photocells for sprint and T-test Marked 30-cm zone for lateral hop tests | Vertical jumps: 3 trials, best retained; unilateral score = sum of best right + left. Horizontal tests: broad jump/single-leg hop with free arms; side-hop tests over 30-cm zone for 10 reps; 3 trials, best retained. Sprint: start 30 cm behind first photocell; 2 trials, best time. Modified T-test: 4 trials (right/left), average of best side-specific efforts. | NR |
| Stern et al.,  2020 | CMJ, SLJ, 10m, 30m sprint, COD | Bilateral and unilateral countermovement jump Bilateral and unilateral broad jump 10- and 30-m sprint 5-0-5 test | Just Jump contact mat Brower Timing Systems gates | CMJ/SLCMJ: hands on hips, self-selected countermovement, 2 trials, 45-s rest, best height from flight time. Broad jump/SLBJ: 'stick' landing for 3 s, 2 trials, best distance. Sprint: 2-point staggered stance 30 cm behind line; 2 trials, 3-min rest, fastest time. 505: 15 m sprint with 180° turn off each leg; 2 trials/leg, timed from 10-m gate to return through gate. | Within-session ICC and CV computed; ranged from moderate to excellent. |
| Cao et al.,  2024 | COD (5-0-5 test) | Unilateral countermovement jump Isometric squat test Knee flexor strength test Single-leg land-and-hold 5-0-5 test | VALD ForceDecks force plates/software Fusion Sport photocells for 5-0-5 | IST/KFS: two 5-s maximal trials per leg; average used. LHT: land from 30-cm platform, 2 trials/leg, average used. UCMJ: single-leg jump on force plate, hands on hips, self-selected depth (~>=30 cm), 2 trials/leg, average peak force. 5-0-5: start 0.25 m before first gate; 2 trials/foot, best time retained and averaged by leg for asymmetry analysis. | Within-study ICCs reported across tests (0.86-0.94). |
| Mujezinović et al., 2024 | 5m, 20m sprint, COD (5-0-5 test) | 505 test 5-m and 20-m sprint | Brower Timing System | Arrowhead: high start, 10 m to center cone, 5 m right/left, diagonal cut to final cone 15 m from start, then return. Sidestep: lateral movement between parallel lines 4 m apart, crossed 6 times without crossing legs. 505: run 15 m, turn at 15-m line; time recorded from 10-m gate out and back. Sprints: high start over 5 m and 20 m. | NR |
| Zhao et al.  2024 | CMJ ,SJ | Countermovement jump Squat jump Single-leg land-and-hold | ForceDecks force plates VALD ForceDecks software | SLLH: stand on force plates 30 cm in front, hands on hips, single-leg jump/land, hold ≥3 s; 3 trials, 30-s rest, average time-to-stabilization and peak landing force. SJ: unloaded squat on force plates; 3 repetitions, 30-s rest, average peak force and max negative displacement. CMJ: two-legged jump on force plates, hands on hips; 3 trials, 30-s rest, average peak power and landing force. | NR |
| Aztarain-Cardiel et al.2025 | CMJ, SLJ 20m sprint, COD (V-cut) | Countermovement jump Horizontal jump 20-m straight-line sprint V-Cut test | Chronojump BoscoSystem contact platform and photoelectric cells Tape measure for HJ | CMJ/CMJ-L/CMJ-R: 3 attempts, 30-s rest, hands on hips; average used if jump spread ≤2 cm, otherwise 2 extra trials and average of middle 3. HJ/HJ-L/HJ-R: 3 attempts, 30-s rest, best distance. Sprint: start 0.5 m behind first gate, 2 trials, best time. V-Cut: 25 m with 4 x 45° turns, 2 trials, 3-min rest, best time. | Study-calculated ICC, SEM and CV reported for measures. |
| Hammami et al.,  2025 | 20m sprint, COD (15m) | 20-m linear sprint Y-Balance Test 15-m change-of-direction with ball | Brower Timing photocells Standardised FIFA size-5 ball and digital pressure gauge | Sprint: start 20 cm behind first gate; gates at 0.4 m height; best of 2 trials. 15-m CoD with ball: 3 m run-in, 3-m slalom through 3 poles, 0.5-m hurdle, then 7-m sprint; 2 familiarisation trials plus recorded trials. | Reference-based reliability reported sprint ICC = 0.99; CoD ICC = 0.94. |
| Zhang & Li,  2026 | CMJ, SLJ, 10m, 20m sprint, COD (5-0-5 test) | Countermovement jump 10-m and 20-m sprint tests 505 agility test Standing long jump Repeated sprint ability test | Certified jump mat or force plate for CMJ Electronic timing gates for sprint/COD/reactive agility Marked floor / tape for SLJ | CMJ: 2 familiarisation + 3 maximal trials, best height used. Sprint: split-stance start through 0/10/20 m gates, 3 trials, 3-min rest, fastest time. 505: 10 m to turn line, 180° pivot, 5 m return, best time each side. Reactive agility: 5 m forward then visual cue to left/right 45° finish gate; 4-6 randomized trials, best time. SLJ: standing start with arm swing and bilateral landing. | Reference-based reliability cited for CMJ, sprint, 5-0-5 test. |

Note. CMJ, countermovement jump; SJ, squat jump; SLJ, standing long jump; COD, change of direction; SCMJ, Single-leg countermovement jump; SCOD, Single-leg change of direction

Table S3. Within-group effect sizes of Unilateral plyometric training on physical fitness outcomes

| **Outcome** | **K** | **SMD**  **(95% CI)** | **I²**  **(%)** | **P**  **(overall effect)** |
| --- | --- | --- | --- | --- |
| CMJ | 7 | 0.47 (0.15 to 0.78) | 15.3 | 0.003 |
| SLJ | 4 | 0.54 (-0.12 to 1.21) | 67.9 | 0.111 |
| Single-leg CMJ | 6 | 1.05 (0.59 to 1.50) | 74.7 | <0.001 |
| ≤10-m sprint | 6 | -0.91 (-1.24 to -0.58) | 0 | <0.001 |
| ≥20-m sprint | 8 | -0.50 (-0.76 to -0.24) | 0 | <0.001 |
| Change-of-direction | 7 | -0.84 (-1.38 to -0.29) | 70.8 | 0.003 |
| Single-leg change-of-direction | 4 | -0.86 (-1.15 to -0.57) | 0 | <0.001 |

Note. CMJ, countermovement jump; SJ, squat jump; SLJ, standing long jump; COD, change of direction; SCMJ, Single-leg countermovement jump; SCOD, Single-leg change of direction

Table S4. Within-group effect sizes of Bilateral plyometric training on physical fitness outcomes.

| **Outcome** | **K** | **SMD**  **(95% CI)** | **I²**  **(%)** | **P**  **(overall effect)** |
| --- | --- | --- | --- | --- |
| CMJ | 7 | 0.48 (0.19 to 0.77) | 0 | 0.001 |
| SLJ | 4 | 0.63 (0.05 to 1.22) | 58.5 | 0.035 |
| Single-leg CMJ | 6 | 0.93 (0.46 to 1.40) | 77 | <0.001 |
| ≤10-m sprint | 6 | -0.68 (-1.03 to -0.32) | 17.0 | <0.001 |
| ≥20-m sprint | 8 | -0.44 (-0.70 to -0.18) | 0 | <0.001 |
| Change-of-direction | 7 | -0.85 (-1.58 to -0.12) | 83.6 | 0.022 |
| Single-leg change-of-direction | 4 | -0.45 (-0.73 to -0.17) | 0 | 0.002 |

Note. CMJ, countermovement jump; SJ, squat jump; SLJ, standing long jump; COD, change of direction; SCMJ, Single-leg countermovement jump; SCOD, Single-leg change of direction

Table S5. Meta-Subgroup Analysis Results.

| **Subgroup** | **Level** | **K** | **n** | **ES (95% CI)** | **p (Overall Effect)** | **I² (%)** | **p (Heterogeneity)** | **p (Subgroup Difference)** |
| --- | --- | --- | --- | --- | --- | --- | --- | --- |
| **≤ 10 m sprint** | | | | | | | | |
| Age | >14.91 | 3 | 70 | -0.13 (-0.61, 0.34) | 0.576 | 0.0 | 0.513 | 0.700 |
|  | ≤14.91 | 3 | 94 | -0.27 (-0.78, 0.23) | 0.294 | 30.6 | 0.237 |  |
| Training duration | ≥8 | 3 | 102 | -0.08 (-0.63, 0.47) | 0.770 | 47.7 | 0.983 | 0.442 |
|  | <8 | 3 | 62 | -0.38 (-0.88, 0.13) | 0.145 | 0.0 | 0.148 |  |
| **≥20 m sprint** | | | | | | | | |
| Age | >14 | 4 | 100 | 0.07 (-0.32, 0.46) | 0.725 | 0.0 | 0.996 | 0.409 |
|  | ≤14 | 5 | 140 | -0.15 (-0.48, 0.19) | 0.390 | 0.0 | 0.882 | 0.409 |
| Training duration | ≥8 | 5 | 148 | -0.10 (-0.43, 0.22) | 0.533 | 0.0 | 0.842 | 0.643 |
|  | <8 | 4 | 92 | 0.02 (-0.39, 0.43) | 0.922 | 0.0 | 0.962 | 0.643 |
| **CMJ** | | | | | | | | |
| Age | ≥15.815 | 4 | 136 | -0.08 (-0.42, 0.26) | 0.648 | 0.0 | 0.960 | 0.866 |
|  | <15.815 | 3 | 94 | -0.03 (-0.44, 0.37) | 0.872 | 0.0 | 0.911 |  |
| Training duration | ≥8 | 3 | 138 | -0.09 (-0.42, 0.25) | 0.610 | 0.0 | 0.947 | 0.803 |
|  | <8 | 4 | 92 | -0.02 (-0.43, 0.39) | 0.925 | 0.0 | 0.958 |  |
| **COD** | | | | | | | | |
| Age | >13.625 | 4 | 108 | 0.29 (-0.19, 0.76) | 0.243 | 34.8 | 0.203 | 0.065 |
|  | <13.625 | 4 | 110 | -0.29 (-0.67, 0.09) | 0.134 | 0.0 | 0.826 |  |
| Training duration | ≥8 | 5 | 148 | -0.04 (-0.54, 0.46) | 0.876 | 54.5 | 0.067 | 0.772 |
|  | <8 | 3 | 70 | 0.06 (-0.41, 0.53) | 0.798 | 0.0 | 0.677 |  |
| **SCMJ** | | | | | | | | |
| Age | <15.125 | 3 | 74 | 0.15 (-0.26, 0.56) | 0.469 | 0 | 0.724 | 0.209 |
|  | >15.125 | 3 | 120 | 0.50 (0.14, 0.87) | 0.007 | 0.0 | 0.467 |  |
| Training duration | ≥8 | 3 | 124 | 0.49 (0.15, 0.82) | 0.803 | 0 | 0.902 | 0.146 |
|  | <8 | 3 | 70 | 0.06 (-0.41, 0.53) | 0.004 | 0.0 | 0.489 |  |

Note.CMJ, countermovement jump; SLJ, standing long jump; COD, change of direction; SCMJ, Single-leg countermovement ju

Figure S1. Leave-one-out sensitivity analysis for CMJ

Figure S2. Leave-one-out sensitivity analysis for SCMJ

Figure S3. Leave-one-out sensitivity analysis for SLJ

Figure S4. Leave-one-out sensitivity analysis for ≤10m

Figure S5. Leave-one-out sensitivity analysis for ≥20m

Figure S6. Leave-one-out sensitivity analysis for COD

Figure S7. Leave-one-out sensitivity analysis for Single-leg COD
